# Intraductal Components of Breast Cancer Spread to the Areola: A Case Report

**DOI:** 10.7759/cureus.107491

**Published:** 2026-04-21

**Authors:** Seiji Hirai, Shoji Oura

**Affiliations:** 1 Department of Surgery, Kishiwada Tokushukai Hospital, Kishiwada, JPN

**Keywords:** areolar recurrence, breast cancer, ductal spread to the areola, mammary ducts toward the areola, non-invasive ductal cancer

## Abstract

Ductal spread of breast cancer sometimes causes nipple recurrence, but very rarely causes recurrence in the areola. A 65-year-old woman visited our hospital for a detailed examination of a newly developed small induration in the right areola, 15 years after undergoing nipple-preserving surgery for luminal-type breast cancer. The patient had also developed contralateral luminal-type breast cancer six years after the first operation and had undergone nipple-preserving surgery again. MRI at the second operation had shown no abnormalities in the right nipple-areolar complex (NAC). On her first visit to our hospital, we palpated a firm induration in the right areola. Ultrasound showed a mass in the areolar skin with suspected dermal disruption. Core needle biopsy showed invasive cancer cells on pathological examination. The patient, therefore, underwent resection of the NAC with adequate safety margins.

Postoperative pathological study showed luminal-type invasive cancer cells mainly growing in the areolar skin with minimal fat invasion, and two solid luminal-type non-invasive ductal cancers (DCISs): one near the areolar skin and the other deeper in the subcutaneous fat tissue around the NAC. The two DCISs had small, discontinuous mammary duct structures around them with linear fibrous components, i.e., the presumed Cooper ligament. The non-malignant mammary duct structures extended toward the areola, and some of them were located closer to the areolar skin than the DCIS focus located near the skin. We, therefore, judged that this areolar recurrence was caused by DCIS that had been present in the mammary ducts extending toward the areola. Breast surgeons should note that the intraductal components of breast cancer can have ductal spread toward the areola and should resect the Cooper ligament when it is identified around the NAC during the operation to avoid this type of areolar recurrence.

## Introduction

Breast-conserving therapy (BCT) has provided significant benefits to many breast cancer patients for at least the past three decades [1]. Small breast cancers have been the primary candidates for BCT, but were often treated with mastectomy when located near the nipple-areolar complex (NAC) due to the possible infiltration of cancer cells into the NAC, either through ductal spread or stromal invasion. MRI, however, has enabled breast surgeons to treat such breast cancers without immediate total mastectomy, instead performing breast-conserving surgery when no cancer cell infiltration of the NAC is observed on preoperative images [2].

While BCT has become more popular in the treatment of early breast cancer, multicentric breast cancer, and large but indolent breast cancer are more often treated not with breast-conserving surgery but with nipple-sparing mastectomy followed by immediate breast reconstruction [3,4]. The vast majority of breast surgeons have recognized that ductal spread is the most important risk factor in determining NAC preservation in breast cancer surgeries. Diagnostic physicians, therefore, have to evaluate ductal spread more precisely to properly decide the surgical options for the target breast cancer.

It is well known that the intraductal components of breast cancer spread continuously or discontinuously in the mammary gland [5]. Therefore, breast surgeons need to resect as much of the mammary gland as possible just beneath the nipple without causing complications to the NAC in the treatment of breast cancer located close to the NAC. Feasible and adequate maximal resection of the subnipple mammary gland plays a crucial role in nipple-sparing mastectomy without adjuvant radiotherapy to the operative field. We report a rare case of areolar recurrence in a patient 15 years after undergoing nipple-sparing mastectomy for luminal-type non-invasive ductal cancer (DCIS), which was presumably caused by the ductal spread of intraductal cancer cell components toward the areola.

## Case presentation

**Figure 1:**
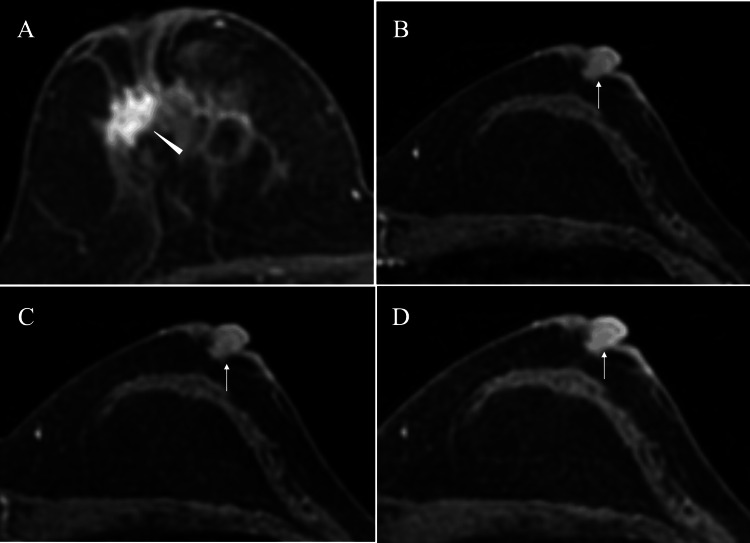
MRI findings at the second operation MRI at the second operation showed non-invasive ductal cancer (arrowhead) in the left breast (A), and no abnormalities in the right nipple-areolar complex (arrow) on fat- suppressed T2-weighted images (B) and on early- (C) and late-phase (D) dynamic study images MRI: magnetic resonance imaging

A 65-year-old woman visited our hospital for a newly developed small areolar induration after nipple-sparing breast cancer surgery. The patient had two children and had exclusively breastfed. She had undergone a right nipple-sparing mastectomy, sentinel node biopsy (SNB), and immediate breast reconstruction using a latissimus dorsi musculocutaneous flap for her estrogen receptor (ER)-positive DCIS 15 years earlier at a hospital. We unfortunately could not retrieve the MRI images due to the expired storage period of the electronic medical record images. We, however, were informed at a later point that at the hospital 15 years earlier, radiologists were creating MRI images not in a standard prone position but in a supine position to obtain images similar to those during breast cancer surgery. MRI at the second operation showed enhancement of the left DCIS and no abnormalities in the right NAC (Figure 1).

**Figure 2:**
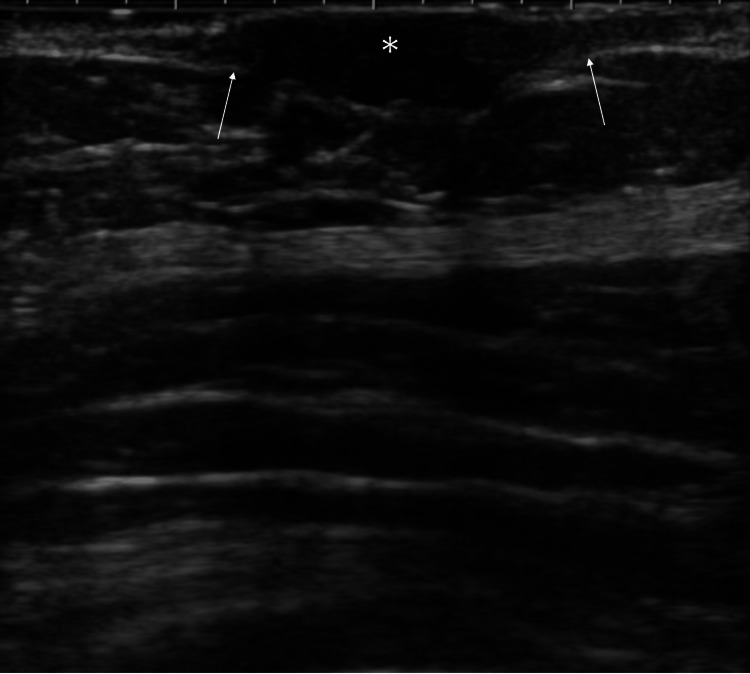
Ultrasound findings Ultrasound showed a mass in the areolar skin (asterisk) which had a hypoechoic pattern, unchanged posterior echoes, and disruption of the dermis (arrows)

Pathological reports of the first operation stated that the nipple base had no cancer cell involvement. The patient had received no radiotherapy to the preserved NAC and completed five years of tamoxifen therapy. She subsequently developed contralateral ER-positive DCIS six years after the first operation and underwent nipple-sparing mastectomy and SNB, without adjuvant radiation therapy. At the second operation, an MRI was performed in a standard prone position and showed no abnormalities in the right NAC. Despite the ER positivity, the patient underwent only semiannual follow-up without adjuvant endocrine therapy after the second operation. Fifteen years after the initial surgery, her attending physician noted an induration in the right areola but only recommended further follow-up, making her anxious and prompting her to visit our hospital to determine whether the areolar induration represented a recurrence. We palpated a small induration in the right areola. Ultrasound of the areolar induration showed a hypoechoic mass mainly located in the skin with partial interruption of the dermis, leading to the diagnosis of an areolar recurrence (Figure 2).

**Figure 3:**
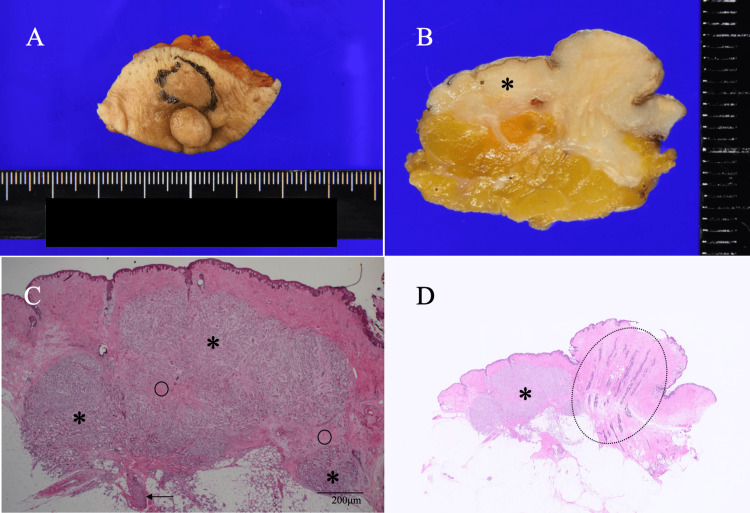
Pathological findings (mainly of macroscopic and low magnified views) A. The recurrent focus (black circle) located in the areola adjacent to the nipple. B. Bisected specimen showed the major part of the recurrence (asterisk) in the areolar skin despite the unclear margins on macroscopy. C. Magnified view showed cancer cells growing in a cord-like fashion (asterisks) with desmoplastic proliferation (open circles) and a solid type non-invasive ductal cancer very close to the areolar skin (arrow) (H.E. ×100). D. Low magnified view showed many mammary ducts (dotted open oval) and the recurrent focus (asterisk) (H.E. ×4)

**Figure 4:**
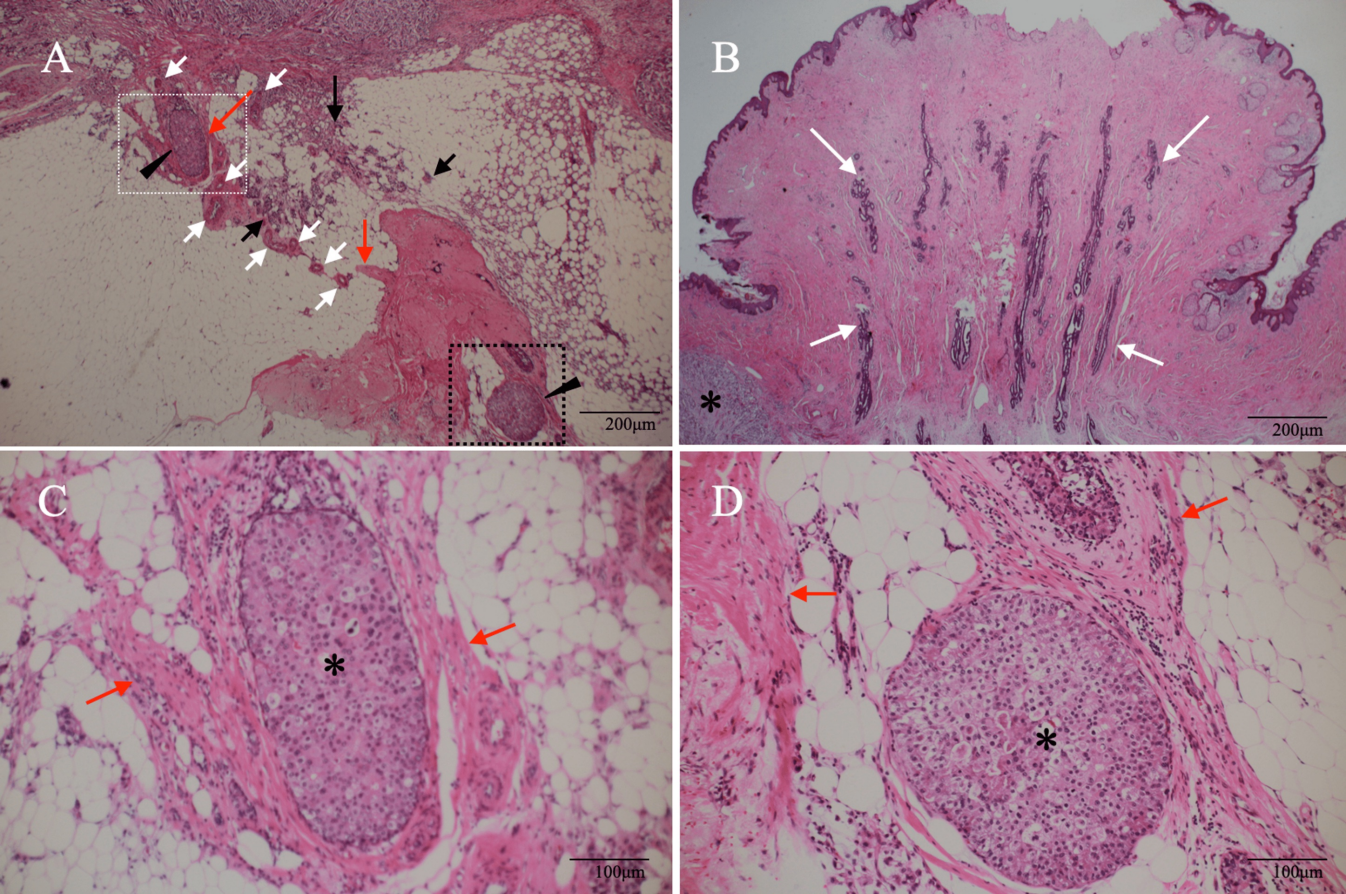
Pathological findings (mainly of magnified views) A. Magnified view showed fat invasion in very limited areas (black arrows) and two solid-type non-invasive ductal cancers (DCISs, black arrowheads). DCISs had connective tissue (red arrows) and mammary duct structures (white arrows) around them (H.E. ×100). B. The resected nipple had many mammary duct structures (white arrows) and no malignant cells (H.E. ×100). C. Magnified view of the white dotted square in panel A showed a solid-type DCIS (asterisk) in the connective tissue (red arrows) (H.E. ×200). D. Magnified view of the black dotted square in panel A also showed a solid-type DCIS (asterisk) surrounded by fibrous connective tissue (red arrows) (H.E. ×200)

Core needle biopsy pathologically showed invasive breast cancer cells growing in a trabecular and solid fashion with a desmoplastic reaction, leading to the pathological diagnosis of areolar recurrence. Immunostaining of the core needle biopsy specimen showed ER positivity (Allred score 8), progesterone receptor positivity (Allred score 6), human epidermal growth factor receptor type 2 (HER2) equivocal, and a high Ki-67 labeling index of 33% [6]. We, therefore, resected the right NAC with safety margins with curative intent. Postoperative pathological study showed a 9 mm mass within the areolar skin, which consisted of cancer cells mainly growing in a trabecular fashion and had minimal invasion into the subcutaneous fat. Pathological examination further showed two solid-type DCIS lesions deep in the area, slightly apart from the region just under the nipple and the area near the areolar skin, respectively, which were accompanied by multiple small mammary ducts extending toward the areola in the linear fibrous components, i.e., the presumed Cooper’s suspensory ligament (Figures 3, 4).

**Figure 5:**
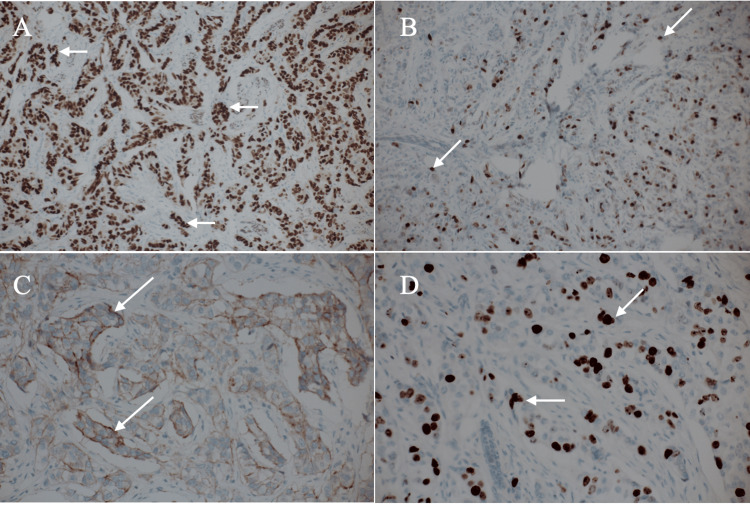
Immunostaining findings Immunostaining showed estrogen receptor positivity (A, arrows; Allred score 8), progesterone receptor positivity (B, arrows; Allred score 6), human epidermal growth factor receptor type 2 equivocality (C, arrows), and a Ki-67 labeling score of 25% (D, arrows)

All immunostaining results were similar to those of the core needle biopsy specimen except for the Ki-67 labeling index of 25% (Figure 5).

**Figure 6:**
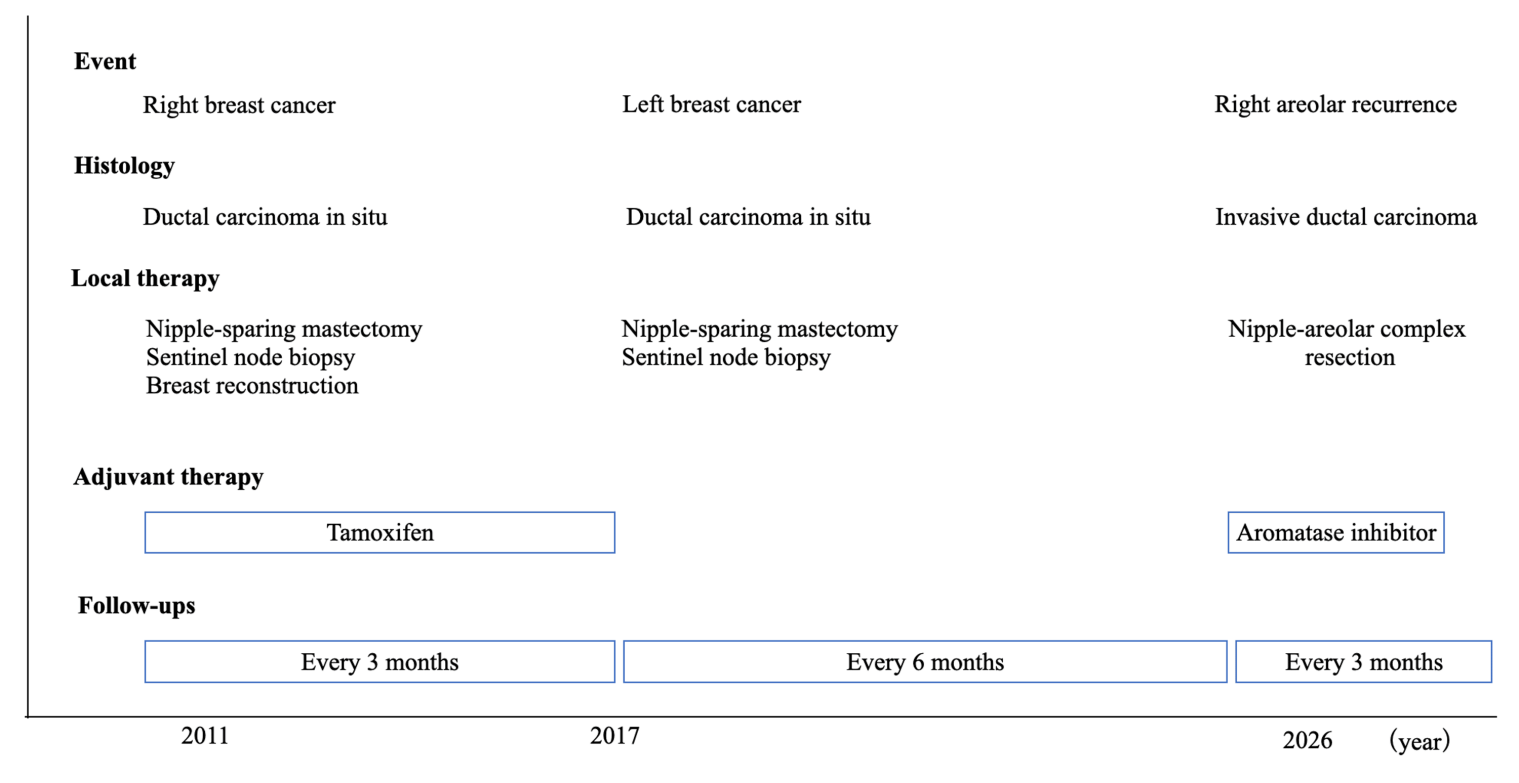
Timeline of the patient’s clinical course

Fluorescence in situ hybridization clarified no gene amplification of HER2. The patient recovered uneventfully, did not receive adjuvant radiotherapy due to surgical clear margins, and is scheduled to be followed up on aromatase inhibitor therapy (Figure 6).

## Discussion

Our patient had a solid-type DCIS deep in the subnipple area, which suggested the possible pathological underestimation of cancer cell spread to the nipple base at the primary surgery. The deep DCIS focus, however, appeared to have no continuity with the nipple. In addition, the two solid DCIS foci had mammary duct structures, showing somewhat different features from those of the intra-nipple mammary ducts around them. The superficial DCIS focus further had mammary duct structures located closer to the skin than those. The presence of fibrous components around the DCIS lesions and the ductal structures strongly suggested that the recurrence in the areola occurred due to the presence of DCIS components within the ductal structures that were originally located within Cooper’s suspensory ligament from the mammary gland to the areola [7].

If mammary duct structures extending toward the areola had been present, they could have produced milk discharge from the areola during lactation. Mammary ducts extending toward the areola, however, lacked continuity, unlike mammary ducts observed in the nipple, suggesting the absence of milk discharge from the areola. In fact, the patient did not experience milk discharge from the areola. In short, this patient had mammary duct structures spreading toward the areola, which seemed nonfunctional as milk passage routes and were unable to discharge milk.

In any case, it is reasonable to conclude that the DCIS that remained dormant within the discontinuous mammary ducts extending toward the areola caused the areolar recurrence in this case. We unfortunately could not retrieve MRI figures before the initial surgery due to the expired storage period of the electronic medical record images, but we were informed by the breast surgeons that the hospital created MRI images in a supine position, similar to the operative position, at the first surgery. This imaging method naturally had lower spatial resolution than the standard prone position method and would have been more affected by respiratory motion, undoubtedly producing poorer images than those obtained in the prone position [8]. On the other hand, even a standard MRI performed before the second operation did not show any abnormalities in the preserved right NAC. It, therefore, seems likely that intraductal cancer cell extension toward the areola in this case would have been impossible to visualize regardless of the quality of the MRI images.

We have reported several times that preservation of drainage veins from the NAC plays a critical role in safe preservation of the NAC. The drainage veins of the nipple are located in the fat tissue just under the skin around the NAC. We, therefore, preserve the subcutaneous fat around the NAC as much as possible for maximal resection of the subnipple and even intra-nipple mammary gland [9-11]. It, therefore, seems reasonable for breast surgeons to resect the mammary gland as extensively as possible using a thick flap method and to completely remove Cooper’s ligaments when identified between the mammary gland and the areola during the operation.

No studies in the literature have evaluated the ductal spread toward the areola to date. Breast surgeons, therefore, should take this type of possible ductal spread to the areola into consideration when preserving NAC. Breast surgeons should further consider this type of areolar recurrence on long-term follow-ups.

## Conclusions

We encountered an exceptionally rare case of local recurrence in the areola, presumably due to cancer cell ductal spread toward the areola 15 years after NAC preservation. Diagnostic physicians and breast surgeons must keep this type of tumor spread in mind when performing imaging evaluations and deciding on surgical options, respectively. In addition, breast surgeons should remove Cooper’s ligament extending from the mammary gland toward the areola when detected during NAC-preserving surgery.
